# Detection of CTC Clusters and a Dedifferentiated RNA‐Expression Survival Signature in Prostate Cancer

**DOI:** 10.1002/advs.201801254

**Published:** 2018-11-15

**Authors:** Molly Kozminsky, Shamileh Fouladdel, Jae‐Seung Chung, Yugang Wang, David C. Smith, Ajjai Alva, Ebrahim Azizi, Todd Morgan, Sunitha Nagrath

**Affiliations:** ^1^ Department of Chemical Engineering University of Michigan 2300 Hayward Street Ann Arbor MI 48109 USA; ^2^ Translational Oncology Program University of Michigan 1600 Huron Pkwy Ann Arbor MI 48109 USA; ^3^ Biointerfaces Institute University of Michigan 2800 Plymouth Road Ann Arbor MI 48109 USA; ^4^ Department of Internal Medicine University of Michigan 1500 E. Medical Center Drive Ann Arbor MI 48109 USA; ^5^ Department of Urology University of Michigan 1500 E. Medical Center Drive Ann Arbor MI 48109 USA; ^6^ Department of Internal Medicine Division of Hematology/Oncology University of Michigan 1500 E. Medical Center Drive Ann Arbor MI 48109 USA

**Keywords:** circulating tumor cells, GO Chip, microfluidics, prostate cancer, RNA expression

## Abstract

Rates of progression and treatment response in advanced prostate cancer are highly variable, necessitating non‐invasive methods to assess the molecular characteristics of these tumors in real time. The unique potential of circulating tumor cells (CTCs) to serve as a clinically useful liquid biomarker is due to their ability to inform via both enumeration and RNA expression. A microfluidic graphene oxide‐based device (GO Chip) is used to isolate CTCs and CTC clusters from the whole blood of 41 men with metastatic castration‐resistant prostate cancer. Additionally, the expression of 96 genes of interest is determined by RT‐qPCR. Multivariate analyses are conducted to determine the genes most closely associated with overall survival, PSA progression, and radioclinical progression. A preliminary signature, comprising high expression of stemness genes and low expression of epithelial and mesenchymal genes, potentially implicates an undifferentiated CTC phenotype as a marker of poor prognosis in this setting.

## 1 Introduction

While men with metastatic castration‐resistant prostate cancer (mCRPC) have a median survival of approximately 18 months, there is substantial heterogeneity, and time‐to‐progression varies widely.^1^ Additionally, given the evolving treatment landscape, there is a clear need for better biomarkers of progression and treatment response in order to help guide therapeutic decisions. While soft tissue and bone biopsies can provide molecular information, many men with mCRPC have already undergone multiple prior invasive biopsies, and the tissue‐based information is representative of only that single disease site.

Circulating tumor cells (CTCs) shed from tumors can be detected in the blood stream^2^ and have the potential to serve as a liquid biopsy. This approach offers the potential for repeated, non‐invasive measurements and may more widely sample the overall disease state. In addition to giving insight into the burden of disease, these cells can relate the overall molecular state and risk of progression through the analysis of gene expression and phenotype of the traveling cells.^3^ However, key obstacles to capturing CTCs include their rarity among the millions of surrounding white blood cells (WBCs) and red blood cells.^4^ To best interrogate CTCs, they must be detected with high yield and sufficient purity.

This problem has been addressed with a host of isolation technologies.^5^ Notably, the first FDA‐approved CTC isolation technology, CellSearch, has been used to establish survival differences based on CTC enumeration.^6^ This macroscale technology uses a magnetic ferrofluid conjugated with an antibody against the epithelial cellular adhesion molecule (EpCAM) to capture EpCAM‐expressing cells from 7.5 mL whole blood.^7^ For increased sensitivity and flexibility of downstream analysis, microfluidics and nanomaterials^8^ have been developed to isolate and study CTCs.^9^, ^10^, ^11^, ^12^

Prior studies have used cell enrichment technologies coupled with reverse‐transcription polymerase chain reaction (RT‐qPCR) to study prostate CTCs and their RNA. For example, mCRPC patients with AR‐V7‐positive CTCs displayed abiraterone and enzalutamide resistance, indicating the potential for liquid biopsy approaches to provide predictive information.^13^ The original microfluidic CTC‐Chip was used to study prostate CTCs in localized and metastatic patients, with CTCs detected in 23/36 metastatic patients.^9^ RT‐qPCR was used to detect the TMPRSS2‐ERG fusion in 9/20 metastatic patients. Next‐generation CTC chips such as the Herringbone (HB) Chip^10^ and the geometrically enhanced differential immunocapture (GEDI) chip^14^ have also been applied to prostate cancer. The HB Chip was used to investigate androgen receptor (AR) signaling through immunofluorescence staining for the prostate specific antigen (PSA) and the prostate specific membrane antigen (PSMA),^15^ while further immunofluorescence characterization by the GEDI chip examined ERG expression.^16^ Recently, the CTC‐iChip was used to reveal the role of noncanonical Wnt signaling through single‐cell RNA‐Seq of prostate CTCs from 13 patients isolated by negative selection.^11^ However, the majority of these studies reported on a limited set of genes.

**Figure 1:**
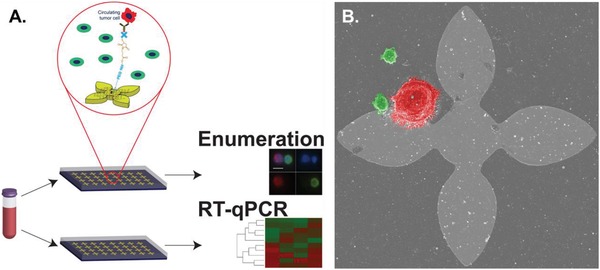
Graphene oxide chip enables isolation of prostate CTCs. A) Sample workflow. Two parallel devices were processed, one each for circulating tumor cell enumeration and RNA extraction. B) Scanning electron micrograph of PC‐3 cell (red pseudocolor) and WBCs (green pseudocolor) on‐chip. Flower patter is 100 µm in height and width.

The nanomaterial‐based graphene oxide chip (GO Chip) affords highly sensitive and gentle capture of rare cells with low WBC contamination.^17^ Optimized with cell line spike‐in samples with as few as 3–5 cancer cells per milliliter of whole blood, the device showed promise in the capture of PC‐3 cells under physiologically relevant conditions and concentrations.^17^ Coupled with the capability for downstream molecular and morphologic analysis, the GO Chip enables CTC enumeration, characterization, or RNA expression from as little as 1 mL whole blood of patient samples.^17^ The efficiency and sensitivity of this device facilitated our study of CTC enumeration and RNA expression in a clinical cohort using an extensive 96 gene panel. Toward the goal of using CTCs to provide clinically relevant molecular information that could eventually be utilized to assist with patient management, we undertook a prospective study of 41 men with mCRPC (**Figure** **1**). We sought to utilize captured CTCs and extracted RNA from parallel GO Chips to determine CTC characteristics associated with progression and survival in advanced prostate cancer.

## 2 Results

### 2.1 Clinical Cohort

Blood samples were collected with informed consent from 41 patients with mCRPC (Table S1, Supporting Information) recruited under institutional approved IRB (HUM00052405) between August 2013 and November 2016 using EDTA tubes. Processing occurred on the day of blood draw. Eight healthy male controls were recruited internally and processed in the same manner as patient samples. The median patient age was 73 years (range: 50–84 years), while the median baseline PSA level was 37.9 ng mL^−1^ (range: 1.2–6433 ng mL^−1^). The median number of prior treatments other than first‐line hormonal therapy was one (range: 0–7), and at the time of CTC collection there were 17 patients receiving abiraterone, four receiving cabazitaxel, two receiving cabozantinib, seven receiving docetaxel, eight receiving enzalutamide, one receiving olaparib, and one receiving pembrolizumab. During the study and follow‐up period, 34 patients experienced PSA progression; 37 experienced radioclinical progression as defined by a ≥20% increase in the sum of the soft tissue lesion diameters during computed tomography, ≥2 new bone lesions on bone scan, or symptomatic progression (worsening pain aggravation or new cancer‐related symptoms); and 22 patients died. For surviving patients, the median time to last follow‐up was 19.1 months (range: 3.3–37.8 months). Median time to death was 17.5 months (range: 2.6–39.6 months).

In addition to overall survival, PSA at the time of blood draw, radiographic, and clinical progression events were recorded. PSA progression was defined using the PCWG3 criteria of an increase of greater than or equal to 25% from the nadir, with a minimum increase of 2 ng mL^−1^.^18^ Radioclinical progression was also used as a clinical endpoint using the date of whichever happened earliest. Radiographic progression entailed one of three events: 20% or more increase in the sum of the diameters of soft‐tissue target lesions based on RECIST criteria applied to CT scans; an increase of at least 5 mm in the short axis of a previously normal lymph node (this lymph node must be at least 1.0 cm in the short axis); or at least two new bone lesions. Clinical progression was defined as worsening disease‐related symptoms or new cancer‐related complications. Radioclinical progression was assessed by a single reviewer using standard PCWG3 criteria.^18^

#### 2.2. Circulating Tumor Cell Detection, Enumeration, and Gene expression Analysis by RT‐qPCR in Clinical Samples

**Figure 2:**
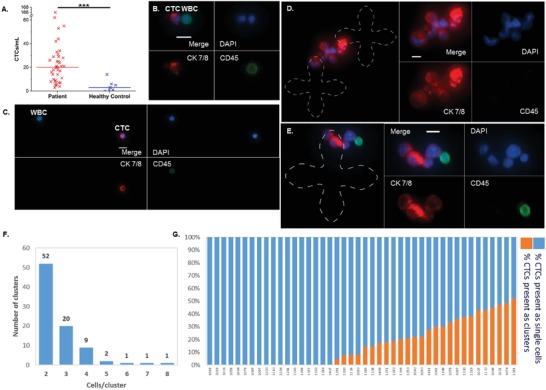
CTCs and CTC clusters isolated by the graphene oxide chip. A) CTC enumeration results for 41 mCRPC patient samples (range: 3–166 CTCs mL^−1^, median: 20) and epithelial cells detected in eight healthy controls (range: 0–14 epithelial cells mL^−1^, median: 3). ***denotes *p* < 0.001. B,C) Examples of CTCs captured on‐chip as well as non‐specifically bound WBCs. Nuclear staining is shown in blue, cytokeratin 7/8 in red, and CD45 in green. D,E) Examples of captured CTC clusters. CTCs captured within clusters had heterogeneous size and cytokeratin expression. The capture pattern is outlined with a dashed line for visualization purposes. Scale bar is 10 µm. F) Captured CTC clusters ranged in size from two to eight cells per cluster. G) The percentage of captured CTCs present in clusters ranged from 0% to 54.8%.

CTCs were detected in all 41 samples with the number of CTCs ranging from 3–166 CTCs mL^−1^ (median: 20 CTCs mL^−1^, **Figure** **2**A–C). The median number of CTCs detected in healthy controls was 3 CTCs mL^−1^ (range: 0–14 CTCs mL^−1^). CTC counts for patients were significantly higher than those for healthy controls (*p* = 0.0001). Quantification of contaminating WBCs is summarized in Table S2 in the Supporting Information.

#### 2.3 Circulating Tumor Cell Cluster Detection in Patient Samples

While processing patient samples, we observed groups of two or more adjacent CTCs (Figure 2D,E), termed CTC clusters. These clusters were only present in patient samples (26/41, 63.4%) and not healthy controls. Both interpatient and intrapatient heterogeneity were evident from the captured clusters, as cells within the clusters showed varying size and cytokeratin expression. Clusters consisted of up to eight CTCs per cluster (Figure 2F) with the majority of the clusters comprising fewer numbers of cells. The percentage of CTCs captured in the form of clusters also varied greatly among patients from 0% to 54.8% (Figure 2G). While CTC clusters have been observed previously in prostate cancer patient samples,^19^, ^20^, ^21^ the high frequency of CTC clusters reported in the present study suggests that the GO Chip may be less disruptive to cell–cell interactions and have greater sensitivity for identifying these clusters.

For 36 of the patients, we had the opportunity to run a parallel microfluidic device that ultimately yielded RNA following cell lysis and purification, which was used for RT‐qPCR (Table S3, Supporting Information). Results from one patient sample were discarded due to insufficient expression of housekeeping genes, suggesting lack of sufficient RNA for analysis. In the remaining 35 patient samples, 77 of the 96 genes were detectable (*C*_T_ < 30) in at least one patient, and 58 genes were detectable at in at least three patients (Tables S4 and S5, Supporting Information). This three‐patient cut‐off was set for two main reasons. The first consideration was to exclude possible technical artifact for genes that show expression only in one to two patients. Second, as much of our subsequent analysis involved comparing relative expression levels between two subgroups of patients, and we wanted to be able to stratify patients into two groups with at least three patients in each subgroup to evaluate.

#### 2.4 Gene‐CTC Enumeration Metric Association

We next examined the relationship between gene expression and CTC counts as well as gene expression and the clusters metrics to determine potential associations. The associations between gene expression and several relevant CTC metrics from each patient sample were examined through linear modeling of these parameters as continuous variables (Figure S1A–F, Supporting Information). If the relationship between the enumeration variable and the gene expression had a negative linear coefficient, the gene expression decreased as the enumeration variable increased (i.e., as the number of CTCs, the number of clusters, or percentage of CTCs in clusters increased). FOXC2 and CTNND1 were positively associated with the number of CTCs mL^−1^ while NFKB1 was negatively associated with CTC counts (*p* < 0.05). CTNND1 and ZEB2 were negatively associated with the number of clusters present per sample, and CTNND1 and FOXC1 were negatively associated with the percentage of CTCs found in clusters (*p* < 0.05). As overexpression of δ‐catenin has been associated with increased proliferation,^22^ the lower expression and therefore potentially lower proliferation would be consistent with an observed stemlike phenotype within the clusters. This is consistent with the lower ZEB2 expression. FOXC1 expression is associated with poor prognosis, androgen independence, and angiogenesis,^23^ potentially indicating aggressive properties in CTCs present as single cells in contrast to those in clusters.

#### 2.5 Multivariate Cox Proportional Hazards Modeling

Multivariate analysis was then conducted using a best subset selection method. As there were 31 PSA progression events, we opted for subsets of two to three genes, applying the “one in ten” rule.^24^ Overall survival, PSA progression, and radioclinical progression were modeled as a function of these two or three subsets using Cox proportional hazards. Models with a global Wald *p‐*value of less than 0.05 were selected and sorted by their Akaike information criterion (AIC),^25^ with lower AICs denoting better quality models. In total, 32 509 combinations were assessed. For overall survival, 311 nominally significant gene combinations (10 two‐gene combinations, 301 three‐gene combinations); for PSA progression, there were 566 nominally significant gene combinations (16 two‐gene combinations, 550 three‐gene combinations); and for radioclinical survival, there were 160 nominally significant combinations (6 two‐gene combinations, 154 three‐gene combinations). Some genes appeared in significant models at higher frequencies than other (Table S6, Supporting Information).

For overall survival, the three‐gene combination of CDH1, CD11B, and STAT3 showed the best performance based on AIC (HR: 0.78, 95% CI: 0.62–0.99; HR: 2.04, 95% CI: 1.32–3.14; and HR: 0.46, 95% CI: 0.30–0.72, respectively). For PSA progression, the three‐gene combination of CD44, CASP3, and FOXC2 performed best (HR: 1.51, 95% CI: 1.23–1.87; HR: 0.79, 95% CI: 0.69–0.90; HR: 0.90, 95% CI: 0.82–0.98, respectively). For radioclinical progression, the best model consisted of the three genes CDH1, AR, and CD45 (HR: 0.84, 95% CI: 0.71–0.98; HR: 1.10, 95% CI: 1.02–1.18; HR: 0.78, 95% CI: 0.66–0.92).

#### 2.6 Exploratory Single Variable Analysis

To assess the clinical relevance of the experimental data obtained, we compared CTC metrics and gene expression with overall survival, PSA progression, and radioclinical progression. None of the enumeration variables, including CTC and cluster counts and related metrics, were statistically significant in the univariable Cox proportional hazards models. However, patients with a high number of clusters relative to the median had a shorter time to radioclinical progression than those with a low number of clusters in the Kaplan–Meier survival analysis (log‐rank *p* < 0.05, Figure S1G, Supporting Information). A previous study associated CTC clusters detected at any of multiple time points with decreased overall survival;^23^ this suggests a future direction for our technology, in which serial sampling may provide more prognostic information.

**Figure 3:**
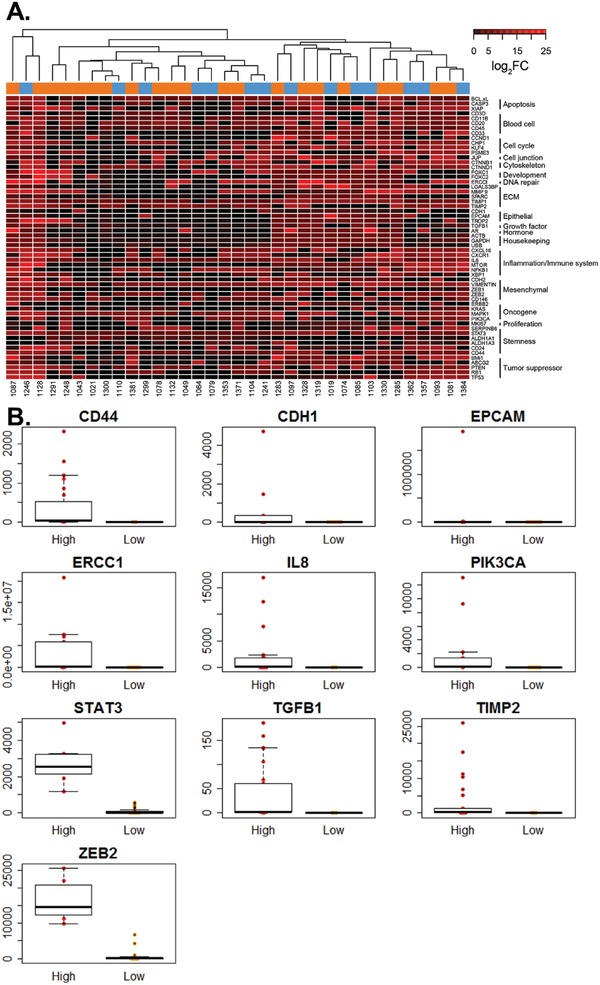
Relative gene expression for use in a bimodal score. A) Heatmap of log_2_ fold changes (FC) relative to healthy control background for 58 genes detected in patient samples. Associated gene categories are shown to the right of the gene list. Color coding above the heatmap indicates the presence (orange) or absence (blue) of clusters in a sample. B) Beeswarm plot of expression (2^−ΔΔ^ *^C^*_t_) for genes enabling stratification based on overall survival.

To construct a bimodal point‐based metric relating gene expression (**Figure** **3**) to clinical outcomes, we used cut‐points to classify patients into high and low survival groups as determined by subsequent Kaplan–Meier analysis. Cut‐points were generated using regression tree analysis from the rpart (recursive partitioning) package in the R software environment. The rpart package uses regression models based on the input data set to find the variable and location that best splits the data into two groups, where best is defined as minimizing the risk of misclassification. In this case, the input data consisted of the gene expression levels and clinical outcomes. The distribution of expression above or below the generated cut‐point was visualized using a beeswarm plot, with expression above the cut‐point denoted as “high” and expression below the cut‐point denoted as “low.” Genes associated with overall survival included CD44, CDH1, EPCAM, ERCC1, IL8, PIK3CA, STAT3, TGFβ, TIMP2, and ZEB2 (Figure 3B). The genes CDH1, CD146, FOXC2, and ZEB2 were associated with PSA progression, while the genes associated with radioclinical progression included ACTB, CDH1, CDH2, CD3D, CD45, CASP3, CD146, CXCR1, KLF4, KRAS, MKI67, MMP9, RB1, SPARC, XBP1, and ZEB2. The number of patients in each group along with the relevant median survival metrics are included in Table S7 in the Supporting Information.

#### 2.7 Multivariate Analysis Derived from Univariate Survival and Progression Analysis

Using these pre‐screened genes, we developed a series of prognostic scores to predict survival/progression. Based on gene expression relative to the cut‐point, we assigned genes a point value of 0 or 1 based on whether their gene expression value fell above or below the cut‐point based on its relationship to shorter survival or progression time. For example, in the case of overall survival, patients expressing greater levels of CD44 than the cut‐point were assigned 1 point, while patients expressing lower levels of CDH1 than the cut‐point were assigned 1 point. Conversely, patients expressing lower levels of CD44 than the cut‐point were assigned 0 points, while patients expressing higher levels of CDH1 than the cut‐point were assigned 0 points. The values for each of the genes were then added in different combinations with all subsets considered and used to generate receiver operating characteristic (ROC) curves, with the scores ranging from 0 to the number of genes in the score. These curves were then evaluated based on the area under the curve (AUC), with AUCs approaching one having greater discriminatory ability. The 95% confidence interval (CI) was generated by resampling, with 10^4^ bootstrap samples. Additionally, the Cox proportional hazard ratio^26^ was calculated for each score.

**Figure 4:**
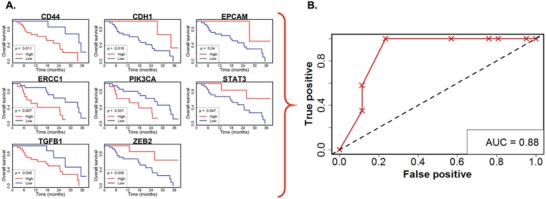
Relationship between RNA expression and overall survival. A) Kaplan–Meier curves for genes with statistically significant relationships with overall survival used to construct the optimized point‐based score. B) Scores were optimized by maximizing the AUC of the associated ROC curve.

Of the significant genes established for overall survival, a set of eight genes ultimately had the best prognostic power: CD44, CDH1, EPCAM, ERCC1, PIK3CA, STAT3, TGFB1, and ZEB2 (**Figure** **4**A). The relative survival of the patients based on the differential expression of these genes is represented in Figure 5A in the Supporting Information. The group with genes expressed at higher levels than the cut‐point is shown in red while the other group below the cut‐point is shown in blue, clearly demonstrating the differences in prognosis between the two groups. The resulting ROC had an AUC of 0.88 (95% CI: 0.69–0.98, Figure 4B). Assessing the score using the Cox proportional hazard model yielded a hazard ratio of 1.83 (95% CI: 1.33–2.51) for overall survival, representing the increased risk between the patient groups one unit apart on the numerical score. Three of the genes in the score, CDH1, EPCAM, and ZEB2, are reflective of epithelial or mesenchymal phenotypes. The association of the low expression of both the epithelial and mesenchymal genes with lower overall survival suggests the importance of a transitory phenotype in leading to disease progression. This potentially undifferentiated phenotype is in concordance with the high expression of prostate cancer stemness marker CD44.^27^ Low ZEB2 was also implicated in the scores for PSA progression and radioclinical progression, while low CDH1 was implicated in the score for PSA progression, suggesting an emerging theme. Additionally, STAT3 is activated by AR signaling loss and is associated with cancer stem cells.^28^ These observations are in line with previous descriptions of stemlike tumor‐propagating subpopulations in prostate cancer.^29^

Using the same methods to relate the subsets of significant genes to radioclinical progression produced a five‐gene score consisting of CD3D, MMP9, RB1, XBP1, and ZEB2 (AUC: 0.82, 95% CI: 0.67–0.96, Figure S2, Supporting Information). This score carried a hazard of 1.51 (95% CI: 1.19–1.92) for radioclinical progression. Finally, investigation of combinations of genes related to PSA progression resulted in a three‐gene score: CDH1, CD146, and ZEB2 (AUC: 0.69, 95% CI: 0.47–0.87, Figure S3, Supporting Information); and a proportional hazard of 1.75 (95% CI: 1.17–2.63).

## 3 Discussion

Overall, our study has important limitations. Without question, this work will continue to benefit from longer follow‐up and corroboration in larger cohorts. Further work to validate this approach and to explore the implications of CTC stemness is ongoing. Our results also highlight the importance of examining protein and RNA expression in CTCs, as we were able to use anti‐EpCAM for capture (indicative of protein expression), but saw low expression of EpCAM RNA. While the previous work establishing the device gives us confidence in our ability to capture low EpCAM‐expressing cells,^17^ future work may incorporate additional capture antibodies to account for CTC heterogeneity. The recruitment of additional controls will be important to examine background levels of cytokeratin positive cells. This false positive rate, a marker of the balance between assay sensitivity and background noise, will need to continue to be refined. Furthermore, our current study makes use of bulk RNA extraction and RT‐qPCR. These techniques were selected to efficiently study the maximum number of genes from a cohort of this size. While we have taken steps to subtract “background” signal from WBCs in the form of processed healthy control samples, we plan to integrate single‐cell techniques into future work to better assess intrapatient heterogeneity and reduce our signal‐to‐noise ratio.

## 4 Conclusion

CTCs provide an opportunity to study the underlying biology and disease trajectory of prostate cancer in a readily‐available liquid biopsy. The incorporation of microfluidics into CTC research offers substantial promise for overcoming the hurdles of low CTC counts and the immense quantity of surrounding normal blood cells. It was our goal to integrate an ultra‐sensitive CTC isolation technology enabling both immunofluorescence characterization and RNA expression analysis in order to investigate the relationship of the CTC‐based results with key oncologic outcomes. Our work here is an example of a clinically relevant biomarker discovery approach utilizing CTC‐related metrics.

## 5 Experimental Section

*Device Fabrication*: Fabrication of the graphene oxide chip (GO Chip) was described previously.^17^ Briefly, a suspension was made by probe tip sonicating graphene oxide (CheapTubes.com) and tetrabutylammonium hydroxide (TBA, Fluka) in dimethylformamide (Sigma‐Aldrich). Phospholipid‐polyethyleneglycol‐amine (PEG, NOF America Corporation) was added to the resulting suspension and bath was sonicated for 1 h. Silicon wafers with gold features fabricated using photolithography were dipped in the suspension to allow self‐assembly of the GO‐TBA‐PEG onto the gold. This pattern was then enclosed in a polydimethylsiloxane (PDMS, Dow Corning) microfluidic chamber. The crosslinker *N*‐γ‐maleimidobutyryl‐oxysuccinimide ester (GMBS, Pierce) was then added to the devices and incubated at which point tubing (Tygon) was inserted. Following a wash, NeutrAvidin (Invitrogen) was added to the devices via syringe pump (Harvard Apparatus). The devices could be stored at 4 °C until use, at which time biotinylated anti‐EpCAM (Table S8, Supporting Information) in 1% bovine serum albumin (BSA, Sigma) was added prior to sample processing.

*Patient Sample Processing*: Following blocking with BSA, 1 mL whole blood was introduced into the GO Chip at a flow rate of 1 mL h^−1^. Devices were then flushed with a total volume of 6 mL phosphate buffered saline (PBS, Gibco) at 100 µL min^−1^ immediately following blood flow. Subsequent steps were determined based on the ultimate application of the device in the work‐flow. For devices that would be stained for enumeration, the contents of the PDMS chamber were fixed using 4% paraformaldehyde (PFA, ThermoFisher). These devices were then stored at 4 °C until they were stained. On a parallel device, RNA extraction was performed by first flowing RNA extraction buffer (a component of the PicoPure RNA isolation kit, Arcturus). The device and syringe were then incubated for 30 min at 42 °C, after which DEPC water (ThermoFisher) was flowed. Collected RNA extraction buffer and DEPC water from the device outlet were stored at −80 °C until purification. Research involving human subjects was performed in accordance with the requirements of the University of Michigan's Human Research Protection Program. Signed and informed consent was obtained from all subjects under institutional approved IRB (HUM00052405).

*Immunofluorescence Staining*: Subsequent to processing and fixation of the sample, immunofluorescence staining was performed on‐chip using a syringe pump. Cells were permeabilized using Triton X (Sigma) and then blocked with a combination of goat serum (ThermoFisher) and BSA. See Table S8 in the Supporting Information for all antibody information. Primary antibodies against CD45 and cytokeratin 7/8 were detected using the appropriate secondary antibodies labeled with Alexa Fluors 488 and 546. Antibodies were suspended in 1% BSA while 2‐(4‐amidinophenyl)‐1*H*‐indole‐6‐carboxamidine (DAPI, Invitrogen) in PBS was used to label cell nuclei. Imaging of fluorescence staining was conducted on a Nikon Eclipse Ti fluorescence microscope using either a 10x or 20x objective. Images were captured using a QImaging cooled mono 12 bit camera and analyzed using NIS‐Elements software. Those nucleated cells expressing CK, but not CD45 (DAPI+/CK+/CD45− cells), were counted as CTCs.

*Quantitative RT‐qPCR*: Bulk cell lysates extracted during sample processing from 36 patients and four healthy controls were subsequently purified using the remaining components of the PicoPure RNA isolation kit; five patients were processed only for enumeration and not RNA expression analysis (Table S3, Supporting Information). Purification was conducted according to the manufacturer's protocol. Purified RNA was reverse transcribed to cDNA according to the manufacturer's protocol using an Ambion kit (ThermoFisher). The cDNA was pre‐amplified after which it underwent RT‐qPCR using an Applied Biosystems TaqMan Gene Expression Assay. Using the BioMark HD qPCR platform (Fluidigm), *C*_T_ levels were determined for 96 genes of interest (complete list, Table S4, Supporting Information) in the following categories: apoptosis, blood cell, cell cycle, cell junction, cytoskeleton, developmental, DNA repair, extracellular matrix, epithelial, growth factor, hormone, housekeeping, inflammation/immune system, long noncoding RNA, mesenchymal, oncogene, proliferation, stemness, transcription factor, and tumor suppressor. To visualize this data, a heatmap was generated using the heatmap function from the “stats” package in the R programming environment. Hierarchical clustering was performed using the complete linkage method as a function of Euclidean distance, which was the default setting for that function.

*Statistical Analysis*: The primary outcome of interest was overall survival. Data variables were related to either enumeration or RNA, and in the case of enumeration included CTCs mL^−1^, the presence of clusters, the number of clusters, the percentage of CTCs in clusters, the average number of CTCs/cluster, and the maximum number of CTCs/cluster. The base ten log or z score of the enumeration variables was taken for the purposes of analysis. Analyses were performed using Excel and R with the following R packages: rpart,^30^ survival,^31^ and survivalROC.^32^ The CTC enumeration variables were compared to the clinical metrics using Cox proportional hazards models and Kaplan–Meier survival analysis. The Wald test was used to determine significance for Cox proportional hazards modeling, while the log‐rank test was used in the Kaplan–Meier analysis. In other comparisons, statistical significance was determined using the Mann–Whitney test. A nominal *p*‐value of less than 0.05 was considered statistically significant. RT‐qPCR results were first normalized to the mean of three housekeeping genes (GAPDH, ACTB, UBB) to obtain a Δ*C*_t_ value, and then background corrected by deducting the mean expression level of each in the four healthy controls to obtain a ΔΔ*C*_t_ value, and subsequently analyzed as log_2_(2^−ΔΔ^ *^C^*_t_ + 1). To select genes for the generation of a point‐based score, analysis conducted with the rpart package in R was used as a screening mechanism, with nominally significant genes being considered. Scores were analyzed using ROC curves using the survival ROC function. As employed in our analysis, this function plotted the true positive rate against the false positive rate for the score's ability to predict median survival time using the nearest neighbor estimation method. A higher AUC from this function denoted better model performance.

## Conflict of Interest

The authors declare no conflict of interest.

## Supporting information

Supplementary
