# Supplementary material for: Detection of CTC Clusters and a Dedifferentiated RNA‐Expression Survival Signature in Prostate Cancer
Source: Adv Sci (Weinh). 2018 Nov 15;6(2):1801254. doi: 10.1002/advs.201801254 (PMC6343066; doi:10.1002/advs.201801254)
Supplement: Supplementary file 1 — Supplementary [file ADVS-6-1801254-s001.pdf]

Copyright WILEY-VCH Verlag GmbH & Co. KGaA, 69469 Weinheim, Germany, 2018.

ADVANCED SCIENCE
Open Access

Supporting Information

for *Adv. Sci.,* DOI: 10.1002/advs.201801254

Detection of CTC Clusters and a Dedifferentiated RNA-Expression Survival Signature in Prostate Cancer

Molly Kozminsky, Shamileh Fouladdel, Jae-Seung Chung, Yugang Wang, David C. Smith, Ajjai Alva, Ebrahim Azizi, Todd Morgan, and Sunitha Nagrath**

Copyright WILEY-VCH Verlag GmbH & Co. KGaA, 69469 Weinheim, Germany, 2016.

Supporting Information

Detection of CTC clusters and a dedifferentiated RNA-expression survival signature in prostate cancer

Molly Kozminsky, Shamileh Fouladdel, Jae-Seung Chung, Yugang Wang, David C. Smith, Ajjai Alva, Ebrahim Azizi, Todd Morgan*, Sunitha Nagrath*

Materials and Methods

Cell culture: The prostate cancer cell line PC-3 cell line was used to generate a scanning electron microscopy image to demonstrate cell capture on the GO Chip. This cell line was a generous gift from the lab of Mark L. Day following their purchase from ATCC. They were maintained in RPMI 1640 (Gibco) supplemented with 10% fetal bovine serum (FBS, Corning) and 1% antibiotic-antimycotic (Gibco). Cells were maintained in a 37°C incubator with 5% CO_2. The cell line tested negative for Mycoplasma contamination as assayed using the Lonza Mycoalert on February 27, 2017.

Scanning electron microscopy: To fix cells on the device for scanning electron microscopy (SEM) imaging, the PDMS layer was removed and the substrate was fixed in 2.5% glutaraldehyde (Electron Microscopy Sciences) for one hour. The substrate was then washed twice with PBS and dehydrated through successive 10 minute dips in increasing concentrations of ethanol (50%, 70%, 95%, 100% twice). The substrate was dipped twice in hexamethyldisilazane (HDMS, Electron Microscopy Sciences) for 10 minutes each and then dried overnight in a chemical hood. The fixed substrate was then sputter coated with carbon and imaged on a FEI Nova 200 Nanolab.

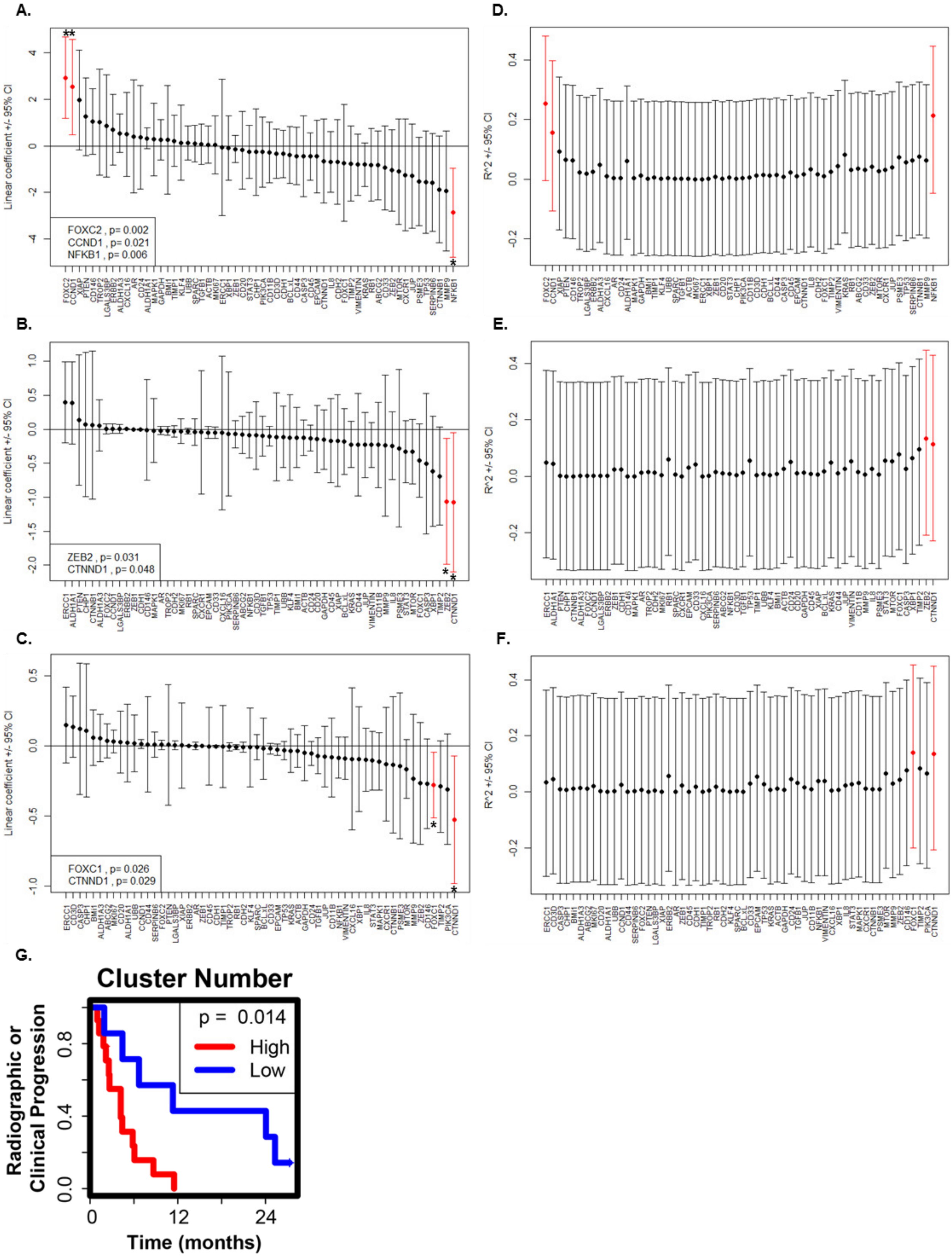

Figure S1. Modeling based on cluster metrics. A) Linear association of gene expression with number of CTCs/mL. Two genes, FOXC2 and CTNND1 showed a significant positive association while NKFB1 showed a significant negative association. B) Linear association of gene expression with number of CTC clusters. Two genes, CTNND1 and ZEB2, showed a significant negative association. C) Linear association of gene expression with percentage of

CTCs in clusters versus present as single cells. FOXC1 showed a significant positive association while CTNND1 showed a significant negative association. D, E, F) R^2 values for linear associations of gene expression with number of CTCs/mL, number of CTC clusters, and percentage of CTCs in cluster versus present as single cells, respectively. Data is plotted as the linear coefficient or R^2 ± the 95% confidence interval; */red denotes $p < 0.05$. G) Relationship between radioclinical progression and number of clusters as determined through Kaplan-Meier analysis ($p < 0.05$).

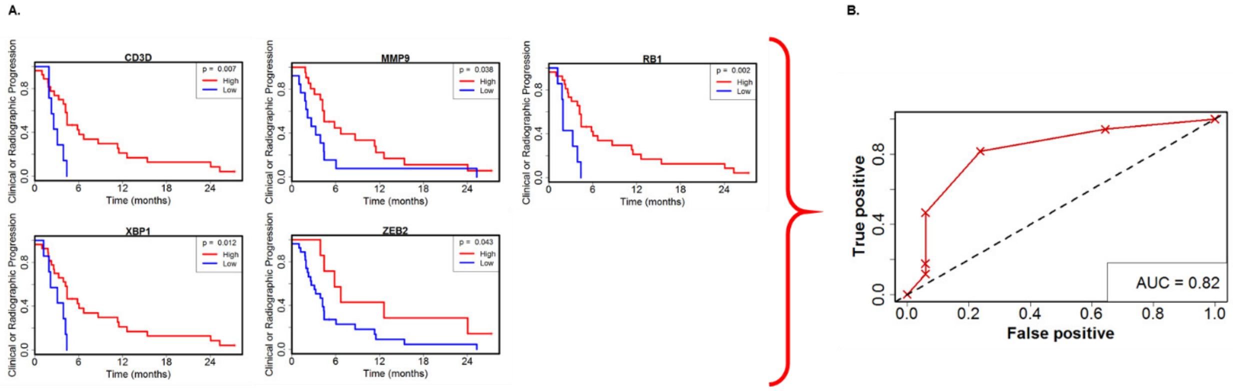

Figure S2. Relationship between RNA expression and radioclinical progression. A) Kaplan-Meier curves for all genes with statistically significant relationships with radiographic or clinical progression used to construct the optimized point-based score. B) Scores were optimized by maximizing the area under the curve (AUC) of the associated receiver operating curve.

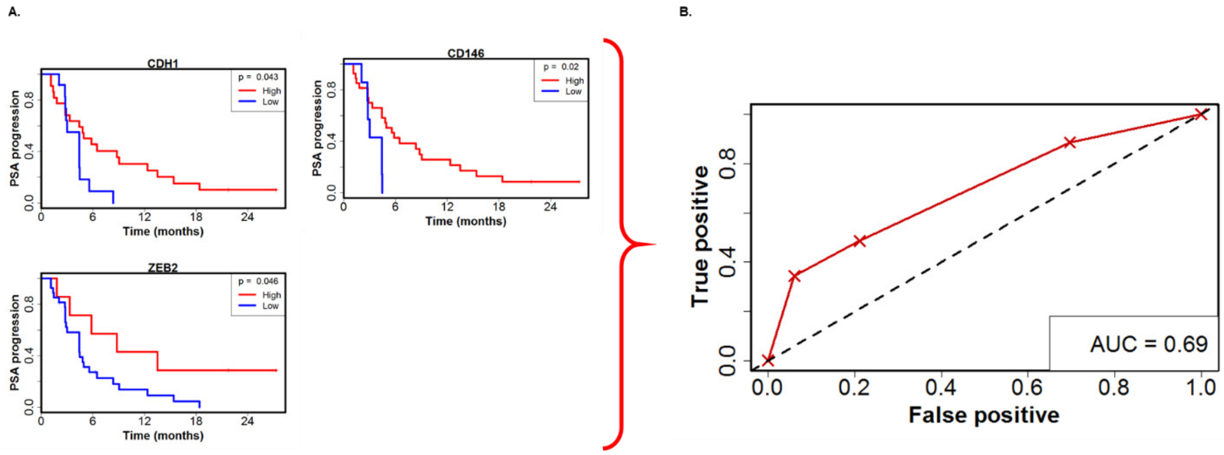

Figure S3. Relationship between RNA expression and PSA progression. A) Kaplan-Meier curves for genes with statistically significant relationships with overall survival used to construct the optimized point-based score. B) Scores were optimized by maximizing the area under the curve (AUC) of the associated receiver operating curve.

Table S1. Clinical cohort information.

Number of patients	41	100%
Age		
Median (range)	73	(50-84)
Race		
Caucasian	39	95.1%
Black	3	7.3%
Castrate resistant	41	100%
Performance status		
0	15	36.6%
1	22	53.7%
2	3	7.3%
Prostate-specific antigen (ng/mL)		
Median (range)	37.9	(1.2-6433.1)
Metastatic disease	41	100%
Visceral metastases	11	26.8%
Number of prior therapies		
Median (range)	1	0-7
Therapy at time of collection		
Abiraterone	17	41.5%
Cabazitaxel	4	9.8%
Cabozantinib	2	4.9%
Docetaxel	7	17.1%
Enzalutamide	8	19.5%
Olaparib	1	2.4%
Pembrolizumab	1	2.4%

Table S2. CTC and WBC counts for patients and healthy controls. A median of 20 CTCs/mL (range: 3-166 CTCs/mL) and 3 epithelial cells/mL (range: 0-14 epithelial cells/mL) were captured from patient and healthy samples, respectively. A median of 437 WBCs/mL (range: 56-54261 WBCs/mL) and 595.5 WBCs/mL (range: 359-2473 WBCs/mL) were non-specifically bound from patient and healthy samples, respectively.

Sample	CTCs/mL	WBCs/mL
1019	20	107
1021	53	264
1043	9	1292
1049	20	2169
1064	3	308
1074	33	482
1078	18	5938
1079	7	56
1081	10	355
1085	11	631
1087	25	690
1092	24	421
1093	18	418
1097	21	499
1103	8	243
1104	16	1034
1110	21	398
1128	25	878
1132	14	54261
1241	5	199
1246	7	344
1248	20	6050
1283	42	411
1285	14	509
1291	44	282
1299	4	199
1300	26	821
1319	29	8394
1328	28	6175
1330	8	341
1353	166	295
1357	24	289
1362	7	437
1371	34	902
1381	21	412
1384	5	10948
1399	55	558
1401	31	259
1407	17	329
1409	29	707
1411	62	1058
HC-1	3	1234
HC-2	14	645
HC-3	5	546
HC-4	5	678
HC-5	1	2473
HC-6	3	392
HC-7	0	454
HC-8	1	359

Table S3. Sample flow through study

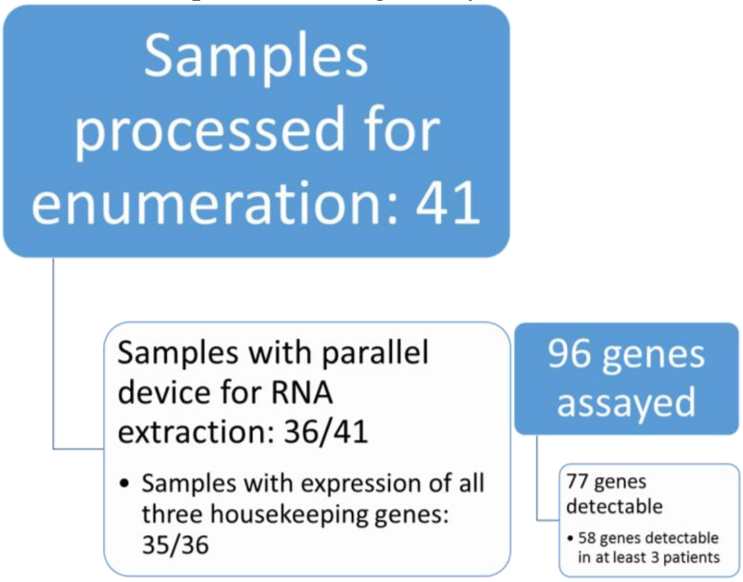

Table S4. List of 96 genes analyzed by RT-qPCR

Gene	Category
**BCL-xL**	Apoptosis
**CASP3**	Apoptosis
**XIAP**	Apoptosis
**CD3D**	Blood cell
**CD11B**	Blood cell
**CD20**	Blood cell
**CD45**	Blood cell
**CD33**	Blood cell
**CD34**	Blood cell
**PTPRN2**	Cell cycle
**CCND1**	Cell cycle
**CHP1**	Cell cycle
**KLF4**	Cell cycle
**PSME3**	Cell cycle
**DSP**	Cell junction
**EVPL**	Cell junction
**JUP**	Cell junction
**CTNNA1**	Cytoskeleton
**CTNNB1**	Cytoskeleton
**CTNND1**	Cytoskeleton
**PKP2**	Cytoskeleton
**FOXC1**	Transcription factor
**FOXC2**	Transcription factor
**ERCC1**	DNA repair
**COL1A2**	ECM
**COL3A1**	ECM
**LGALS3BP**	ECM
**MMP2**	ECM
**MMP9**	ECM
**SPARC**	ECM
**TIMP1**	ECM
**TIMP2**	ECM
**CDH1**	Epithelial
**EPCAM**	Epithelial
**KRT7**	Epithelial
**KRT8**	Epithelial
**KRT14**	Epithelial
**KLK3**	Epithelial
**EMP2**	Epithelial
**MUC1**	Epithelial
**TROP2**	Epithelial
**FGF18**	Growth factor
**IGFBP5**	Growth factor
**TGFB1**	Growth factor
**AR**	Hormone
**GAPDH**	Housekeeping
**ACTB**	Housekeeping
**UBB**	Housekeeping
**CXCL16**	Inflammation/Immune system
**CXCR1**	Inflammation/Immune system
**IL6**	Inflammation/Immune system
**IL8**	Inflammation/Immune system
**MTOR**	Inflammation/Immune system
**NFKB1**	Inflammation/Immune system
**PD-1**	Inflammation/Immune system
**PDL-1**	Inflammation/Immune system
**XBP1**	Inflammation/Immune system
**XIST**	lncRNA

**HOTAIR**	lncRNA
**CDH2**	Mesenchymal
**CDH11**	Mesenchymal
**VIMENTIN**	Mesenchymal
**KRT5**	Mesenchymal
**SNAI1**	Mesenchymal
**SNAI2**	Mesenchymal
**ZEB1**	Mesenchymal
**ZEB2**	Mesenchymal
**CD146**	Mesenchymal/endothelial
**ERBB2**	Oncogene
**ERG**	Oncogene
**FOLH1**	Oncogene
**ALK**	Oncogene
**EGFR**	Oncogene
**KRAS**	Oncogene
**MAPK1**	Oncogene
**NTRK2**	Oncogene
**PIK3CA**	Oncogene
**MKI67**	Proliferation
**MLPH**	Signal transduction
**SERPINB6**	Signal transduction
**STAT3**	Signal transduction
**ALDH1A1**	Stemness
**ALDH1A3**	Stemness
**CD24**	Stemness
**CD44**	Stemness
**CD44v6**	Stemness
**CD133**	Stemness
**BMi1**	Stemness
**ABCG2**	Stemness/Drug resistance
**ETV1**	Transcription factor
**TMPRSS2**	Transcription factor
**ELF3**	Transcription factor
**TP53**	Tumor suppressor
**PTEN**	Tumor suppressor
**RB1**	Tumor suppressor
**PTCH1**	Tumor suppressor

Table S5. Detected genes

Genes detected in one or more patients (C_T < 30)	Detected in three or more patients?
**ABCG2**	Yes
**ACTB**	Yes
**ALDH1A1**	Yes
**ALDH1A3**	Yes
**AR**	Yes
**BCL-xL**	Yes
**BMi1**	Yes
**CASP3**	Yes
**CCND1**	Yes
**CD11B**	Yes
**CD146**	Yes
**CD20**	Yes
**CD24**	Yes
**CD33**	Yes
**CD3D**	Yes
**CD44**	Yes
**CD45**	Yes
**CDH1**	Yes
**CDH2**	Yes
**CHP1**	Yes
**CTNNB1**	Yes
**CTNND1**	Yes
**CXCL16**	Yes
**CXCR1**	Yes
**EPCAM**	Yes
**ERBB2**	Yes
**ERCC1**	Yes
**FOXC1**	Yes
**FOXC2**	Yes
**GAPDH**	Yes
**IL8**	Yes
**JUP**	Yes
**KLF4**	Yes
**KRAS**	Yes
**LGALS3BP**	Yes
**MAPK1**	Yes
**MKI67**	Yes
**MMP9**	Yes
**MTOR**	Yes
**NFKB1**	Yes
**PIK3CA**	Yes
**PSME3**	Yes
**PTEN**	Yes
**RB1**	Yes
**SERPINB6**	Yes
**SPARC**	Yes
**STAT3**	Yes
**TGFB1**	Yes
**TIMP1**	Yes
**TIMP2**	Yes
**TP53**	Yes
**TROP2**	Yes
**UBB**	Yes
**VIMENTIN**	Yes
**XBP1**	Yes
**XIAP**	Yes
**ZEB1**	Yes
**ZEB2**	Yes

**CD44v6**	No
**DSP**	No
**EGFR**	No
**ELF3**	No
**EMP2**	No
**ETV1**	No
**EVPL**	No
**FOLH1**	No
**IGFBP5**	No
**IL6**	No
**KLK3**	No
**KRT8**	No
**MLPH**	No
**MUC1**	No
**PDL-1**	No
**PKP2**	No
**SNAI1**	No
**TMPRSS2**	No
**XIST**	No

Table S6. Top ten genes appearing in multivariate Cox proportional hazards models. Frequency is reported as fraction of models for respective survival/progression metrics with global Wald p value of less than 0.05.

Overall survival		PSA progression		Radioclinical progression	
Gene	Frequency	Gene	Frequency	Gene	Frequency
ZEB2	0.49	CHP1	0.36	CASP3	0.43
ERCC1	0.30	SPARC	0.22	SPARC	0.38
CD44	0.26	ZEB2	0.21	CD44	0.37
PTEN	0.20	CD44	0.15	CCND1	0.33
IL8	0.15	CASP3	0.15	CDH1	0.18
CASP3	0.13	FOXC1	0.13	ZEB2	0.15
STAT3	0.11	PIK3CA	0.13	ALDH1A1	0.08
VIMENTIN	0.10	FOXC2	0.13	VIMENTIN	0.06
CD11B	0.09	XBP1	0.13	CD45	0.06
TIMP2	0.09	ACTB	0.12	IL8	0.06

Table S7. Breakdown of patients in high and low gene expression groups. Highlighted genes indicate those genes incorporated into gene scores.

	Gene	High expression	Low expression	Median PSA (ng/mL, High expression)	Median PSA (ng/mL, Low expression)	Median survival time (months, High expression)	Median survival time (months, Low expression)	Median no. prior therapies (High expression)	Median no. prior therapies (Low expression)
Overall survival	CD44	28	7	34.3	40.6	21.3	34.3	1	0
	CDH1	9	26	40.6	30.2	34.3	18.4	1	0.5
	EPCAM	8	27	32.9	37.5	27.3	21.3	0.5	1
	ERCC1	13	22	14.6	50.1	12.8	33.7	1	0.5
	IL8	26	9	35.2	37.45	21.3	34.3	0.5	1
	PIK3CA	13	22	14.6	40.6	17.7	27.3	1	0
	STAT3	7	28	30.2	37.5	N/A	21.3	0	1
	TGFB1	26	9	33.6	171.3	18.4	27.3	1	0
	TIMP2	25	10	18.95	97.85	18.4	34.3	0	1
	ZEB2	7	28	30.2	38.3	N/A	21.3	0	1

	Gene	High expression	Low expression	Median PSA (ng/mL, High expression)	Median PSA (ng/mL, Low expression)	Median progression free survival time (months, High expression)	Median progression free survival time (months, Low expression)	Median no. prior therapies (High expression)	Median no. prior therapies (Low expression)
PSA progression	CDH1	22	13	35.9	27.65	5.38	4.43	0	1
	CD146	28	7	40.6	11.7	5.6	3	0	1
	FOXC2	7	28	54.6	34.3	18.4	4.43	1	0.5
	ZEB2	7	28	30.2	38.3	8.8	4.43	0	1

	Gene	High expression	Low expression	Median PSA (ng/mL, High expression)	Median PSA (ng/mL, Low expression)	Median progression free survival time (months, High expression)	Median progression free survival time (months, Low expression)	Median no. prior therapies (High expression)	Median no. prior therapies (Low expression)
Radioclinical progression	ACTB	20	15	37.9	14.35	4.43	2.92	0.5	1
	CDH1	17	18	37.5	17	6.7	3.27	0	1
	CDH2	19	16	38.3	17	5.83	3.27	0	1
	CD3D	28	7	37.5	14.1	4.43	2.57	1	0
	CD45	25	10	35.9	28.8	4.43	2.25	0	1
	CASP3	27	8	37.5	17	4.43	3.27	1	0.5
	CD146	27	8	45.35	9.25	4.43	2.72	0	1
	CXCR1	28	7	38.3	11.7	4.43	3.27	0	1
	KLF4	22	13	37.9	14.35	5.13	2.3	0	1
	KRAS	28	7	37.5	17	4.43	2.57	1	0
	MKI67	22	13	39.05	15.55	4.43	3.18	0.5	1
	MMP9	22	13	38.3	17	5.83	2.67	0	1
	RB1	28	7	37.5	17	4.43	1.93	0	1
	SPARC	15	20	37.5	34.3	5.83	3.27	0	1
	XBP1	28	7	34.3	38.3	4.43	3.1	0	1
	ZEB2	7	28	30.2	38.3	6.7	3.93	0	1

Table S8. Antibody information.

**Antibody**	**Conjugate**	**Application**	**Company**	**Catalog number**	**Clone**
Goat anti-human EpCAM	Biotin	Cell capture	R&D Systems	BAF960	Polyclonal
Mouse IgG2a anti-human cytokeratin 7/8	N/A	Primary antibody	BD Biosciences	349205	CAM5.2
Mouse IgG1 anti-human CD45	N/A	Primary antibody	BD Biosciences	555480	HI30
Goat anti-mouse IgG2a	Alexa Fluor 546	Secondary antibody	ThermoFisher	A-21133	Polyclonal
Goat anti-mouse IgG1	Alexa Fluor 488	Secondary antibody	ThermoFisher	A-21121	Polyclonal
